# War Service Badges for Hospital Staffs

**Published:** 1915-06-19

**Authors:** 


### THE EDITOR'S LETTER-BOX.

### War Service Badges for Hospital Staffs.

*To the Editor of* The Hospital.

Sir,—It may interest you to know how this hospital has got over the difficulty of the above. Two of our porters have been, and are, constantly being confronted by recruiting officers, who refuse to believe them when they say they are employed in a hospital.

This week we communicated with the officer commanding the military hospitals of this district, who replied that he could not give us permission to issue badges to our men. We communicated with the chief recruiting officer of this district and asked his permission to issue badges, and he replied that he could not give this permission, but suggested that a note should be given to each man certifying that he is a hospital employee, what his work is, and that he cannot be spared for military duties, and that this note should be signed by the secretary and should be shown by the porter to anyone who asks him why he is not in the Army.

We also communicated with the War Office in London, and they replied that they could not give authority for the issue of badges.

Quite on their own account and responsibility, these porters have obtained from a firm in Birmingham a badge, at a cost of 1s. 6d. each, and they intend to wear these and see what the result will be. At the same time they are carrying a letter, signed by myself, certifying what their duties are.—Yours faithfully,

"Hospital Secretary."

